# Unravelling the expression of interleukin-9 in chronic rhinosinusitis: A possible role for *Staphylococcus aureus*

**DOI:** 10.1186/s13601-020-00348-5

**Published:** 2020-10-19

**Authors:** Tim Delemarre, Natalie De Ruyck, Gabriele Holtappels, Claus Bachert, Elien Gevaert

**Affiliations:** 1 Department of Head & Skin, Upper Airways Research Laboratory, Faculty of Medicine, Ghent University, C. Heymanslaan 10, 9000 Ghent, Belgium; 2 Division of ENT Diseases, CLINTEC, Karolinska Institute, Stockholm, Sweden; 3 International Airway Research Center, First Affiliated Hospital, Sun Yat-Sen University, Guangzhou, China

**Keywords:** Chronic rhinosinusitis with nasal polyps, Interleukin-9, *Staphylococcus aureus*

## Abstract

Chronic rhinosinusitis with nasal polyps (CRSwNP) is a Th2 biased inflammation, associated with nasal colonization of *Staphylococcus* (*S.*) *aureus*. Interleukin (IL)-9 is a pro-inflammatory Th2 cytokine with a pivotal role in asthma, allergy and chronic obstructive pulmonary disease (COPD), but is less studied in CRSwNP. We aimed to characterize the expression and cellular source of IL-9 and examined *S. aureus* as potential local trigger in CRSwNP. We showed increased numbers of interleukin-9 producing neutrophils and mononuclear cells in the tissue of CRSwNP patients. This interleukin-9 production was stimulated by *S. aureus* and its enterotoxin B in vitro. These findings underline the contribution of *S. aureus* and define IL-9 as another relevant cytokine in type 2 CRSwNP.

To the Editor

Chronic rhinosinusitis with nasal polyps (CRSwNP) is a chronic Th2 biased inflammation of the sinonasal mucosa, with patients suffering facial pressure, nasal obstruction and reduced smell caused by the presence of nasal polyps and excessive sticky mucus production [1]. CRSwNP is associated with nasal colonization of *Staphylococcus* (*S.*) *aureus* in 67% of the patients [2]. *S. aureus* can infiltrate the nasal mucosa and modulate the CRSwNP pathogenesis via secretion of enterotoxins (Toxic shock syndrome toxin-1 (TSST-1), *S. aureus* enterotoxin A and B (SEA and SEB)), which causes an excessive stimulation of T-cells through their superantigen function. More specifically, *S. aureus* contributes to the polarization of type 2 immune responses in CRSwNP, characterized by increased levels of Th2 cytokines as interleukin (IL)-4, IL-5 and IL-13 [3]. IL-9 is another pro-inflammatory Th2 cytokine that plays a pivotal role in multiple chronic airway inflammations as asthma, chronic obstructive pulmonary disease (COPD) and allergy, but is less studied in CRSwNP [4]. In asthmatic animal models and in vitro experiments, IL-9 causes bronchial hyperresponsiveness, lung eosinophilia, elevated levels of IgE, increased mucin secretion and subepithelial accumulation of collagen – all major characteristics of the CRSwNP pathogenesis [5–7]. In addition, concentrations of IL-9 in bronchoalveolar lavage correlate with clinical parameters of bronchoconstriction in asthmatic patients [6]. Despite the clear effects of IL-9 on other respiratory diseases, studies on IL-9 in CRSwNP are rather limited, and cellular sources, triggers of IL-9 production and possible functions in CRSwNP are still unidentified. Therefore, we aimed to characterize the expression of IL-9 and IL-9R, and made use of *S. aureus* as local trigger of IL-9 production in the tissue of CRSwNP patients. Details of methods and materials used in this study are shown in Additional file 1: Materials and methods. Patient characterization of the subjects used in this study is summarized in Additional file 2: Table S1.

Both *lL9 and IL9R* gene expression were found significantly increased in CRSwNP (n = 40) compared to control (n = 20) tissues (p < 0.05 and p < 0.001; Fig. 1 a, b). As IL-9 protein levels in the tissue were undetectable via ELISA tests, further confirmation of increased IL-9 in CRSwNP was provided by quantification of IL-9 + cells via immunohistochemistry (IHC) staining. Indeed, numbers of IL-9^+^ cells were found significantly increased in CRSwNP (n = 13) compared to controls (n = 5) (p < 0.01; Fig. 1c). These findings are in line with an earlier study in Caucasian patients showing the increased presence of IL-9^+^ cells in CRSwNP, while to the best of our knowledge, this is the first report describing increased IL-9R expression in Caucasian CRSwNP [8]. In addition, we found significantly elevated numbers of IL-9^+^ cells in CRSwNP patients with comorbid asthma (n = 8), compared to those without comorbid asthma (n = 5) (p < 0.05; Additional file 3: Fig. S1). No association was observed between local IL-9 production and the patients’ allergy status (data not shown). Related to these observations, it was shown that numbers of IL-9-expressing cells are increased in asthmatic airways compared to healthy controls and in bronchial biopsies of asthmatic patients with nasal polyps compared to those without nasal polyps [6].

**Fig. 1:**
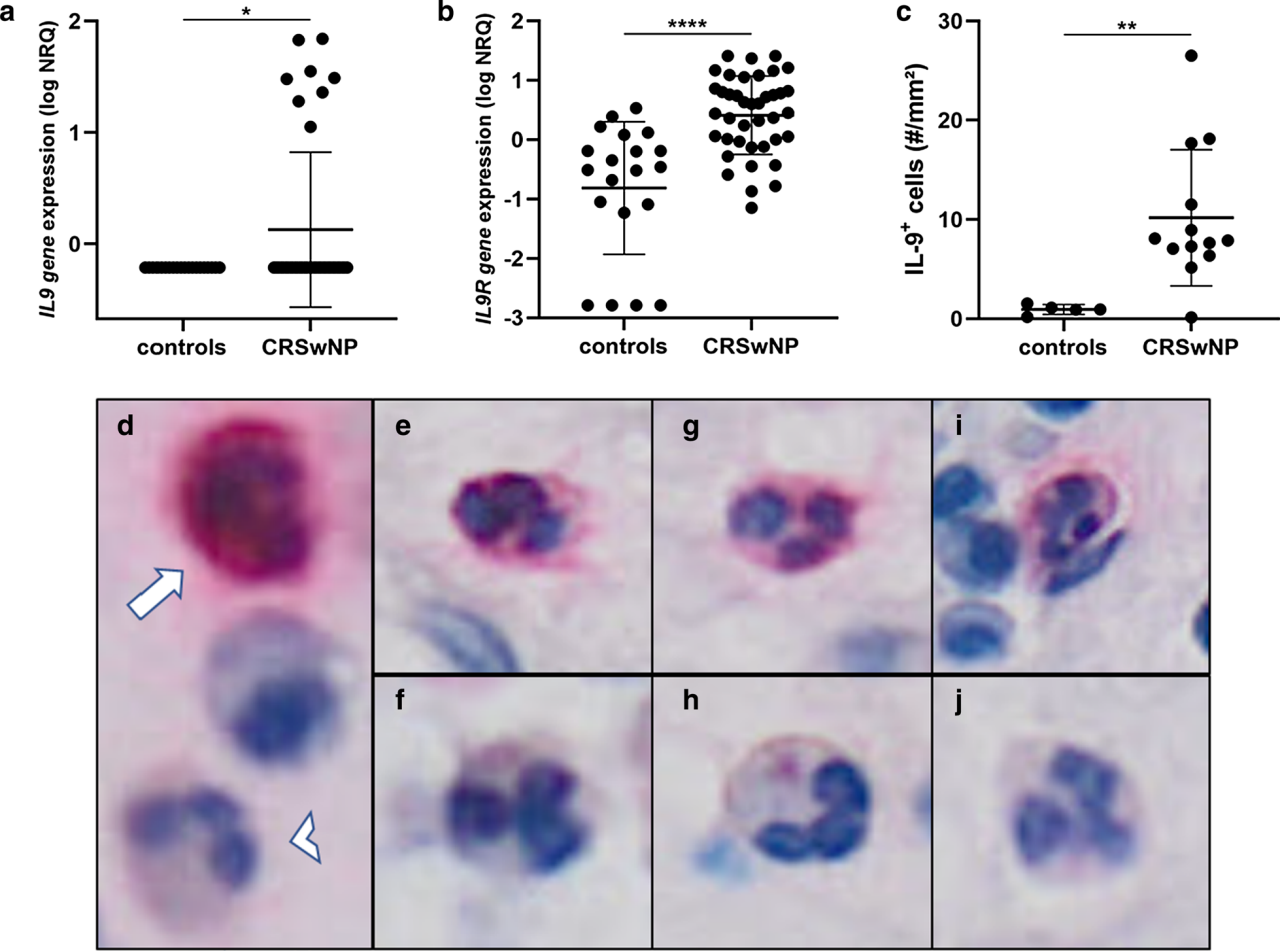
*IL9*–*IL9R* gene expression and numbers of IL-9^+^ cells are increased in CRSwNP compared to controls. **a** *IL9* and **b** *IL9R gene* expression were significantly (resp. p < .05 and p < .0001) increased in tissue of CRSwNP (n = 40) compared to controls (n = 20). **c** Significantly (p < .01) increased numbers of IL-9^+^ cells were observed in tissue of CRSwNP (n = 13) compared to controls (n = 5). Gene expression is expressed as normalized relative quantity (NRQ). **d** IL-9^+^ neutrophil (arrow) and IL-9^−^ neutrophil (arrowhead) located near each other in the same patient. Both IL-9^+^ neutrophils (**e**, **g**, **i**) and IL-9^−^ neutrophils (**f**, **h**, **j**) were observed in patients with CRSwNP. Levels of statistical significance are expressed as *p < .05, **p < .01, ***p < .001 and ****p < .0001

Morphologic analysis of the IHC stainings further showed that, similar to asthmatic models, the main producers of IL-9 in CRSwNP were mononuclear cells and neutrophils [4]. Interestingly, only a fraction (80.1 ± 14.0%) of the neutrophils were IL-9 positive (Fig. 1d–j), which might indicate that IL-9 production in neutrophils is subtype, micro-environment or activation dependent. Besides classic triggering of the T-cell receptor, CD4( +) T-cells require an additional stimulus to produce and secrete IL-9 [7]. CRSwNP pathology is affected by *S. aureus* and its enterotoxins as described above, but the effect of *S. aureus* and SEB on IL-9 production is still unknown. We therefore stimulated peripheral blood derived mononuclear cells (PBMCs) with vehicle (NS), lipopolysaccharide (LPS), *S. epidermidis*, *S. aureus* and SEB for 24 h and analyzed the *IL9 gene* expression in 7 independent experiments. Significant increases in *IL9 gene* expression were observed in PBMCs upon stimulation with *S. aureus* (p < 0.001) and SEB (p < 0.01), but not with *S. epidermidis* and LPS (Fig. 2). Moreover, the increase in *IL9 gene* expression in PBMCs was concentration dependent for *S. aureus* (Additional file 4: Fig. S2). Interestingly, *S. aureus* is a very specific trigger for IL-9 production as no increase was observed upon stimulation with the same concentration of *S. epidermidis*. These observations are in line with earlier findings from our group showing that *S. aureus* colonization is associated with type 2 immune responses and that SEB can induce the production of several type 2 cytokines as IL-4, IL-5 and IL-13 in CRSwNP [2, 9]. To confirm our findings, CRSwNP tissue cubes were stimulated with *S. aureus* and SEB, but due to low RNA quality after stimulation, and the absence of a SEB-compatible ELISA kit, we were unable to measure IL-9 production ex vivo. Altogether, we showed here that the increased levels of IL-9 in CRSwNP are produced by a fraction of neutrophils and mononuclear cells and that the IL-9 production in mononuclear cells is stimulated by *S. aureus* and its enterotoxin B. The increased levels of IL-9 in CRSwNP could play a vital role in the chronicity and severity of CRSwNP, but further research will be necessary to show the exact relevance of IL-9 in CRSwNP. These findings underline the contribution of *S. aureus* to the CRSwNP pathogenesis, and define IL-9 as another relevant cytokine, upregulated with type 2 cytokines such as IL-4, IL-5, IL-13 and others, which deserves further study.

**Fig. 2:**
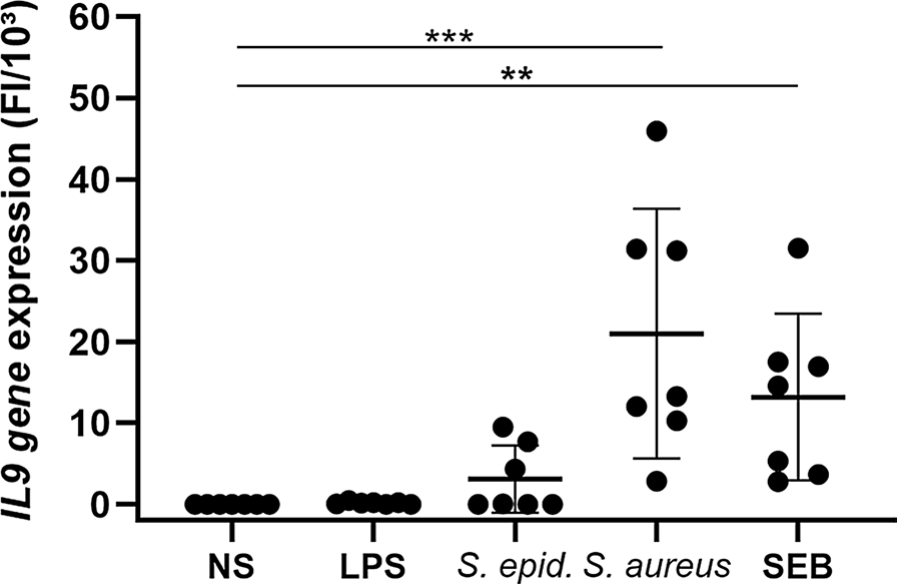
*S. aureus* induced *IL9 gene* expression in PBMCs. Levels of *IL9 gene* expression in PBMCs after 24 h stimulation with culture medium (NS), LPS (1 µg/ml), *S. epidermidis* (10^5^ CFUs/ml), *S. aureus* (10^5^ CFUs/ml) and SEB (1 µg/ml) in n = 7 independent experiments. *IL9 gene* expression in significantly increased upon 24 h stimulation with *S. aureus (*p < .001) and SEB (p < .01), but not upon stimulation with *S. epidermidis* and LPS. Levels of gene expression are expressed as fold induction (FI) versus non stimulated conditions, divided by 10^3^. Levels of statistical significance are expressed as *p < .05, **p < .01, ***p < .001 and ****p < .0001

## Supplementary information

**Additional file 1.** Materials and methods.

**Additional file 2: Table S1.** Patient characterization.

**Additional file 3: Figure S1.** Numbers of nasal tissue IL-9^+^ cells associated with comorbid vs. no asthma. Numbers of IL-9^+^ cells are significantly increased in CRSwNP patients with comorbid asthma (n = 8), compared to those without asthma (n = 5). Patients were considered asthmatic based on their clinical records or diagnosis by a pneumologist. Levels of statistical significance are expressed as *p < 0.05, **p < 0.01, ***p < 0.001 and ****p < 0.0001.

**Additional file 4: Figure S2.** Concentration dependency of IL9 gene expression of PBMCs on S. aureus. Significantly (p < 0.05) increased *IL9 gene* expression when PBMCs were stimulated with 100-fold numbers of *S. aureus* (n = 7). Levels of statistical significance are expressed as *p < 0.05, **p < 0.01, ***p < 0.001 and ****p < 0.0001.

## Data Availability

All data generated or analyzed during this study are included in this published article and its additional files.

## Abbreviations

CRSwNP: Chronic rhinosinusitis with nasal polyps
*S. aureus*: *Staphylococcus aureus*
*S. epidermidis*: *Staphylococcus epidermidis*
TSST-1: Toxic shock syndrome toxin 1
SEA: *Staphylococcus aureus* enterotoxin A
LPS: Lipopolysaccharide
SEB: *Staphylococcus aureus* enterotoxin B
IL: Interleukin
COPD: Chronic obstructive pulmonary disease
IgE: Immunoglobulin E
PBMCs: Peripheral blood mononuclear cells
