# Supplementary material for: Unravelling the expression of interleukin-9 in chronic rhinosinusitis: A possible role for Staphylococcus aureus
Source: Clin Transl Allergy. 2020 Oct 19;10:41. doi: 10.1186/s13601-020-00348-5 (PMC7597062; doi:10.1186/s13601-020-00348-5)
Supplement: Supplementary file 1 — Additional file 1. Materials and methods. [file 13601_2020_348_MOESM1_ESM.docx]

**Methods:**

1. **Sample collection**

At the Department of Otorhinolaryngology at Ghent University Hospital (Belgium), tissue and blood samples from CRSwNP patients (n=53) and healthy patients (inferior turbinate; n=25) undergoing surgery for anatomical obstruction were collected after receiving written informed consent for inclusion. Tissue samples were either snap-frozen or fixated in 4% paraformaldehyde and embedded in paraffin. For the isolation of mononuclear cells from peripheral blood (PBMCs), 100 ml blood was obtained from healthy volunteers and processed within 20 minutes after collection. Clinical data and symptom surveys were collected for all patients that were enrolled in the study (Table E1). Patients were considered asthmatic based on their clinical records or diagnosis by a pneumologist, and allergy was defined as present when the patient had a positive skin prick test for at least one of the allergens commonly tested in our region. Patients who used oral- or intranasal glucocorticosteroids within 4 weeks before surgery were excluded from this study. The study was approved by the local Ethics Committee of Ghent University Hospital (B670201939934).

1. **Cytokine measurements**

Snap frozen tissues were weighed, homogenized and centrifuged as described before. (Ref. E1) Tissue levels of IL-5, IL-17 and TNFα were measured using commercially available Luminex kits from R&D Systems, and IFN-γ using a commercially available Quantikine ELISA from R&D Systems (Minneapolis, Minnesota, USA). Tissue levels of IgE and *S. aureus* enterotoxin (SEA, SEC, TSST-1) specific IgE (SE-IgE) and eosinophil cationic protein (ECP) were measured using the UniCAP method (Thermo Fisher Scientific, Phadia AB, Uppsala, Sweden) according to the manufacturer’s instructions. Concentrations below detection limit were considered negative and were given the value half of the detection limit.

1. **Immunohistochemistry**

Tissues were fixed in 4% paraformaldehyde, embedded in paraffin and slides of 4 µm were prepared. After deparaffination, rehydration and blocking, the slides were incubated with primary antibody (Polyclonal rabbit anti-human interleukin-9; MBS127547; MyBioScource) and isotype control. An alkaline phosphatase linked secondary antibody kit (REAL Detection System, Alkaline Phosphatase/ RED, Rabbit/Mouse; Dako, Agilent Technologies, Diegem, Belgium) was applied following manufacturer’s instructions. Slides were counterstained with hematoxylin and mounted with Aquatex^TM^ mounting medium (Merck). Slides were analyzed as numbers of IL-9^+^ cells/mm² tissue.

1. **RT-qPCR**

RNA was extracted from stimulated PBMCs (section *PBMC stimulations*) and snap-frozen tissue samples of CRSwNP patients (n = 40) and controls (n=20) as described before. (Ref. E2) In summary, RNA was isolated using the RNeasy Mini Kit (QIAGEN, Antwerp, Belgium), cDNA was synthesized using the iScript Advanced cDNA Synthesis Kit for RT-qPCR (Bio-Rad, Temse, Belgium). Quantitative real-time PCR was used to quantify mRNA levels of *IL9* and *IL9R*. The primers were commercially purchased from Bio-Rad. The expression of 2 reference genes, elongation factor 1 (EF-1) and succinate dehydrogenase complex flavoprotein subunit A (SDHA), was used to normalize for transcription and amplification variations among samples after a validation with geNorm (Biogazelle, Zwijnaarde, Belgium). The normalized relative quantities (CNRQs) were calculated with the qBase+ software (Biogazelle, Zwijnaarde, Belgium).

1. **PBMC stimulations**

Peripheral blood mononuclear cells (PBMCs) were isolated from whole blood from healthy volunteers via Ficoll-Paque gradient centrifugation. 10^6^ PBMCs were stimulated for 24h with *S. aureus* (10^5^ CFUs), *S. epidermidis* (10^5^ CFUs), SEB (1 µg/ml), LPS (1 µg/ml) or vehicle in RPMI (Life Technologies) supplemented with 100 U/mL penicillin and 100 mg/mL streptomycin (Life Technologies) and 0.1% BSA (Sigma-Aldrich, Overijse, Belgium). After 24h, PBMCs were washed twice and subsequently lysed with RTL buffer of RNeasy Mini Kit (QIAGEN) prior to RNA extraction.

1. **Statistical analysis**

Statistical analysis was performed with the Prism Graphpad version 8 software program. A Mann-Whitney U test was used to evaluate statistical differences between two groups. A Kruskal-Wallis test, followed by a multiple comparison test was used to evaluate statistical differences between multiple groups. Data was presented in dot-plots for a better representation of interindividual variation. P-values less than or equal to 0.05 were considered as statistically significant. Levels of significance were expressed as *p < 0.05, **p < 0.01, ***p < 0.001 and ****p < .0001.

**Extra references:**

E1. Zhang N, Van Crombruggen K, Holtappels G, Lan F, Katotomichelakis M, Zhang L, et al. Suppression of cytokine release by fluticasone furoate vs. mometasone furoate in human nasal tissue ex-vivo. PLoS One. 2014;9(4):e93754.

E2. Gevaert E, Zhang N, Krysko O, Lan F, Holtappels G, De Ruyck N, et al. Extracellular eosinophilic traps in association with <em>Staphylococcus aureus</em> at the site of epithelial barrier defects in patients with severe airway inflammation. Journal of Allergy and Clinical Immunology. 2017;139(6):1849-60.e6.
