# Supplementary material for: Unravelling the expression of interleukin-9 in chronic rhinosinusitis: A possible role for Staphylococcus aureus
Source: Clin Transl Allergy. 2020 Oct 19;10:41. doi: 10.1186/s13601-020-00348-5 (PMC7597062; doi:10.1186/s13601-020-00348-5)
Supplement: Supplementary file 2 — Additional file 2: Table S1. Patient characterization. [file 13601_2020_348_MOESM2_ESM.docx]

|  |  | **Controls** | **CRSwNP** |
| --- | --- | --- | --- |
| **Total cases** | (#) | 25 | 53 |
| **Gender** | F/M | 6/19 | 18/35 |
| **Age** | Median (range) | 34 (20 - 68) | 45 (18 - 81) |
| **Ethnicity** |  | Caucasian | Caucasian |
| **Allergy** | (+/-) | 12/12 | 34/19 |
|  | missing cases (#) | 1 | - |
| **Asthma** | (+/-) | 4/21 | 29/24 |
|  | missing cases (#) | - | - |
| **Tissue concentrations (Mean ± SD)** |  |  |  |
| **IgE** | U/g | 66.56 ± 76.81 | 932.26 ± 1329.99 |
| **SE-IgE** | UA/g | 1.07 ± 2.72 | 4.16 ± 5.43 |
| **IL-5** | pg/g | 4.84 ± 10.21 | 537.32 ± 680.13 |
| **ECP** | µg/g | 0.46 ± 0.67 | 41.29 ± 123.88 |
| **IL-17** | pg/g | 52.27 ± 81.37 | 42.59 ± 89.58 |
| **TNF-α** | pg/g | 7.99 ± 15.51 | 22.99 ± 23.88 |
| **IFN-γ** | pg/g | 21.45 ± 0.00 | 54.37 ± 86.67 |
