# Supplementary figures and images for: Unravelling the expression of interleukin-9 in chronic rhinosinusitis: A possible role for Staphylococcus aureus
Source: Clin Transl Allergy. 2020 Oct 19;10:41. doi: 10.1186/s13601-020-00348-5 (PMC7597062; doi:10.1186/s13601-020-00348-5)

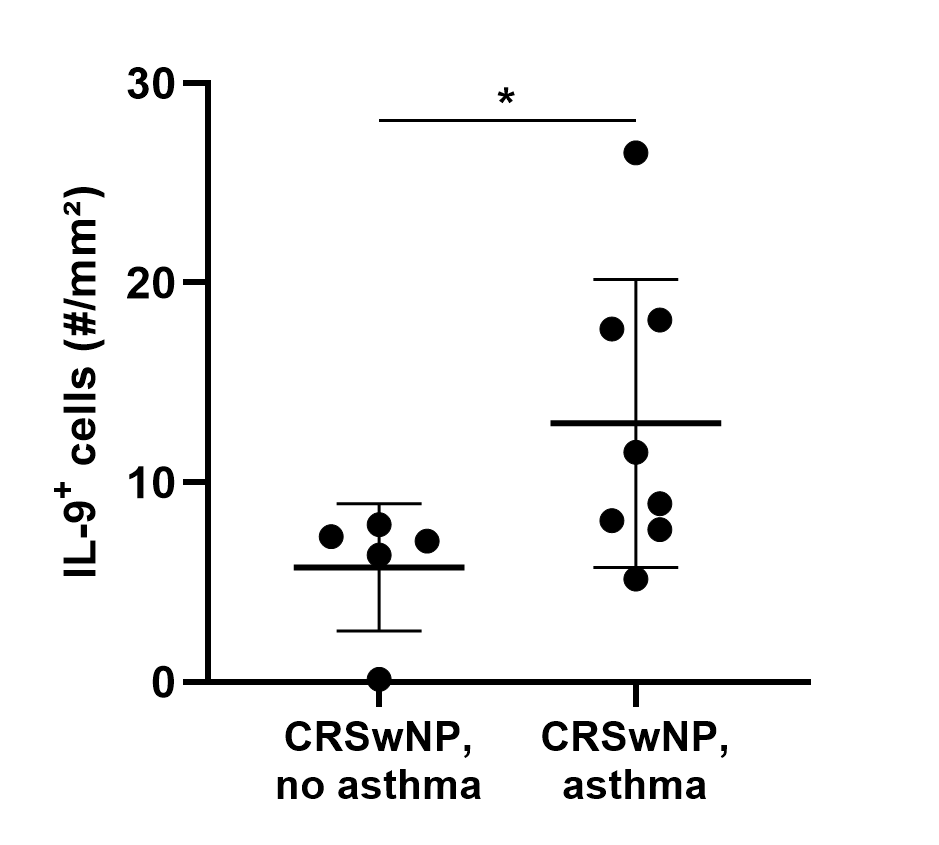

Supplement: Supplementary file 3 — Additional file 3: Figure S1. Numbers of nasal tissue IL-9+ cells associated with comorbid vs. no asthma. Numbers of IL-9+ cells are significantly increased in CRSwNP patients with comorbid asthma (n = 8), compared to those without asthma (n = 5). Patients were considered asthmatic based on their clinical records or diagnosis by a pneumologist. Levels of statistical significance are expressed as *p < 0.05, **p < 0.01, ***p < 0.001 and ****p < 0.0001. [file 13601_2020_348_MOESM3_ESM.tif]

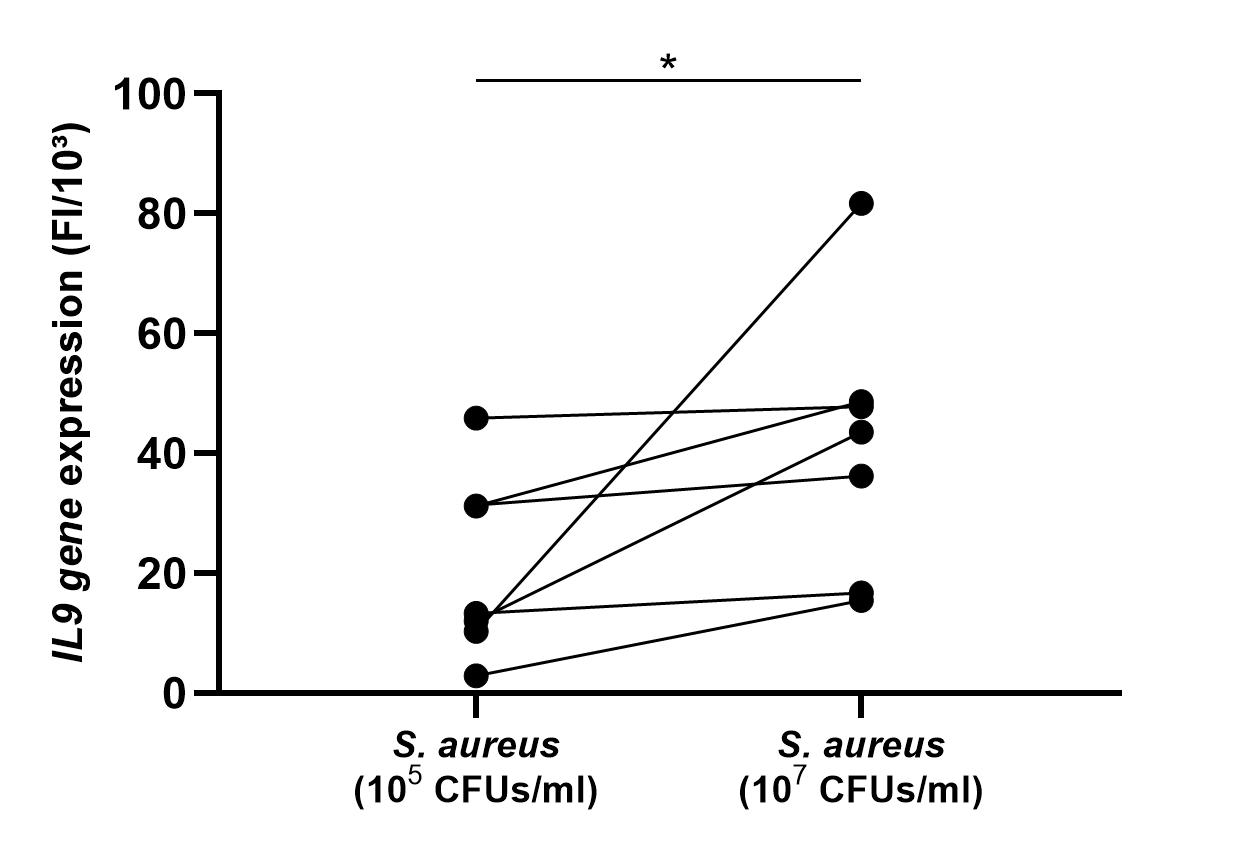

Supplement: Supplementary file 4 — Additional file 4: Figure S2. Concentration dependency of IL9 gene expression of PBMCs on S. aureus. Significantly (p < 0.05) increased IL9 gene expression when PBMCs were stimulated with 100-fold numbers of S. aureus (n = 7). Levels of statistical significance are expressed as *p < 0.05, **p < 0.01, ***p < 0.001 and ****p < 0.0001. [file 13601_2020_348_MOESM4_ESM.jpg]
